# Genetic diversity and structure of an endangered medicinal herb: implications for conservation

**DOI:** 10.1093/aobpla/ply021

**Published:** 2018-03-29

**Authors:** Soo-Rang Lee, Ji-Eun Choi, Byoung-Yoon Lee, Jeong-Nam Yu, Chae Eun Lim

**Affiliations:** 1 Multidisciplinary Genome Institute, Life Science Hall, Hallym University, Hallymdaehak-gil, Chuncheon-si, Gangwon-do, South Korea; 2 National Institute of Biological Resources, Seo-gu, Incheon, South Korea; 3 Nakdonggang National Institute of Biological Resources, Donam, Sangju-si, Gyeongsangbuk-do, South Korea

**Keywords:** *Aconitum*, *austrokoreense*, chloroplast haplotypes, endangered species, gene flow, microsatellite, population structure

## Abstract

Human-driven habitat fragmentation leads to spatial isolation of endangered plant species increasing extinction risk. Understanding genetic variability and population structure of rare and isolated plant species is of great importance for assessing extinction risk and setting up conservation plans. *Aconitum austrokoreense*, an endangered and endemic species in Korea, is a perennial herb commonly used for medicinal purposes. We used five nuclear microsatellites and one chloroplast marker to investigate genetic diversity and population structure for 479 individuals of *A*. *austrokoreense* from seven populations throughout South Korea. A multivariate approach, discriminant analysis of principal components analysis, revealed broad-scale spatial patterns of *A*. *austrokoreense* populations across three major mountains that were composed of seven genetically distinct subgroups. High pairwise *F*_ST_ values (mean *F*_ST_ = 0.35; highest *F*_ST_ = 0.55) suggested significant differentiation among populations. Overall within population genetic variation was low. Based on Mantel test, there was significant correlation between geographical and genetic distances indicating pattern of isolation by distance. Our results suggest that *A*. *austrokoreense* populations may have undergone recent population bottlenecks. Given the limited dispersal ability of the species and ongoing habitat fragmentation, population isolation may further be exacerbated leading to increased extinction risk.

## Teaser

Human-driven habitat fragmentation leads to spatial isolation of endangered plant species, increasing extinction risk. An endangered medicinal herb, *Aconitum austrokoreense*, is a species with limited dispersal ability. Our molecular analysis revealed that within-population level genetic diversity is low, whereas the among-population divergence is high in this species. Moreover, several populations of *A. austrokoreense* might have experienced recent bottlenecks. The results suggested that the species might have declined and/or still decline in population size, resulting in increased extinction risk. Coupled with the life history traits such as dispersal and pollination, recent habitat fragmentation may further increase the extinction risk of the species.

## Introduction

Anthropogenic activities have altered over one-third of land surfaces on earth, resulting in severe habitat destruction (Young *et al.* 1996; Vitousek *et al.* 1997; Lienert 2004). Habitat fragmentation poses major threats to endangered plant species by reducing population size and increasing geographic isolation (Young *et al.* 1996). Given that the increased extinction risk of many endangered species is often associated with small and isolated populations, population genetics is highly relevant to conservation of endangered species (Ellstrand and Elam 1993; Ottewell *et al.* 2015). Population genetics may play a significant role for conservation in several ways including the following. First, small and isolated populations may have reduced genetic diversity due to increased genetic drift and inbreeding (Wright 1932; Ellstrand and Elam 1993; Dlugosch and Parker 2008; Méndez *et al.* 2014; Ellegren and Galtier 2016). For example, based on analysis of 14 microsatellite markers with 36 populations of the Dupont’s lark (*Chersophilus duponti*), genetic diversity and inbreeding appeared to be strongly influenced by size of populations (Méndez *et al.* 2014). Additionally, the population size and variation in floral traits that are heritable were negatively correlated in 16 populations of the endangered plant, *Hypericum cumulicola* (Oakley 2015). Second, limited gene flow may drive small isolated populations to be extirpated (Slatkin 1987; Ellstrand and Elam 1993; Frankham 2015) by inhibiting local adaptation (Bolnick and Nosil 2007).

*Aconitum austrokoreense* is a perennial herb, endemic to Korea that is restricted to southern part of the Korean peninsula (Kadota 1987; Park *et al.* 2016). Since 1993, *A. austrokoreense* has been listed as an endangered species in Korea (Suh and Kim 2012). Aconite roots have widely used as a folk medicine throughout East Asia due to the poisonous alkaloids extracted from the roots, which have greatly raised extinction risks of the species (Lee 1979; Bessonova *et al.* 1988; Singhuber *et al.* 2009; He *et al.* 2010; Tang *et al.* 2012). For example, ~800000 individual plants of *Aconitum heterophyllum*, an endangered herb occurring in Eastern Himalaya, are collected from wild populations each year (Rai *et al.* 2000). In Korea, dried roots of *Aconitum* species are collectively called ‘Buja’ and have been commonly used in oriental medical practices (Yun *et al.* 2015). Although there is no credible number formally investigated, it is likely that along with other species in the same genus, *A. austrokoreense* has been a target of poaching for a long time in Korea (Park *et al.* 2016). Furthermore, the species only inhabits rocky slopes along the mountain valleys (Kadota 1987; Park *et al.* 2016). Given the limited distribution along montane valleys, populations are often small and isolated (Park *et al.* 2016). Poaching for folk remedy and habitat destruction by anthropogenic activities might have led to drastic decline in natural populations of the species (Suh *et al.* 2004; Yoo *et al.* 2005). Despite the urgent need of conservation strategy for the species, knowledge of population dynamics that may greatly contribute to the population decline is very limited.

In this study, we investigated genetic diversity and the spatial pattern of genetic structure of *A. austrokoreense*, an endangered endemic species in Korea. We used microsatellite markers that were successfully isolated for *A. austrokoreense* (Yun *et al.* 2015). We chose microsatellite markers (simple sequence repeats: SSRs) because they are one of the most commonly used molecular markers due to its abundance, codominance, analytically simple and highly polymorphic behaviour (Weber 1990; Selkoe and Toonen 2006; Lopez *et al.* 2015). We aimed to (i) assess the level of genetic diversity in endangered *A. austrokoreense*; (ii) evaluate genetic isolation by geographical distance; (iii) estimate genetic bottlenecks in small and isolated populations; and (iv) propose directions useful for management and recovery plans based on the geographic distribution of genetic diversity. Given that the population sizes are expected to be small with population bottlenecks, we hypothesized that the genetic diversity would be low. We also hypothesized that the populations of *A. austrokoreense* might have greatly diverged over space due to the population isolations derived from biological nature of the species, i.e. specific habitat preferences and limited seed dispersal.

## Methods

### Study species

*Aconitum austrokoreense* (Ranunculaceae) belongs to the subgenus *Aconitum* sect. *Flagellaria* ser. *Racemulosa*. Generally, *Aconitum* species have protandrous flowers that can produce ~10 to 20 minute seeds (ca. 20 mm long) from ellipsoidal follicles (Kadota 1987; Oh and Park 1998; Chung and Park 2000). Solitary bees such as bumble bees are the primary pollinator for the species (Brink 1980; Bosch and Waser 1999; Wang *et al.* 2017). However, flowers are self-compatible when the species are subjected to selfing (Oh and Park 1998). Mature seeds normally fall near the mother plant by gravity, thus the seeds do not migrate far from the mother plant (Kadota 1987). Although a few species of *Aconitum* reproduce through vegetative propagation (Brink 1980; Kadota 1987), there is no evidence that *A. austrokoreense* propagates clonally.

### Sample collection and DNA isolation

A total of 479 individuals (30–187 per population; mean number of samples collected per population = 55; Table 1) were collected from all seven populations throughout the Korean peninsula known from field survey during early autumns of 2013 and 2014 (Table 1). Within each population, collected samples were separated by at least 10 m to avoid multiple samples of possible clones. As the species is an endemic and critically endangered species in South Korea, we received all necessary permits from the Ministry of Environment of Korea prior to sampling. We collected fresh leaves and preserved them at room temperature in plastic zip lock bags with silica-gel desiccant until DNA extraction. Genomic DNA was extracted from dried leaves using the DNeasy Plant Mini Kit (Qiagen, Hilden, Germany) following the manufacturer’s protocol. We measured the quantity and assessed purity of genomic DNA using NanoDrop ND1000 (Thermo Fisher Scientific; quality cut-off, OD 260/280 ratio between 1.7 and 1.9). The extracted DNA was stored at −20 °C until its further use.

**Table 1.** Sampling sites and molecular diversity assessed from nuclear microsatellite markers and a single chloroplast marker; *N*, number of samples collected; *P*, percentage of polymorphic loci; *N*_A_, allelic richness (mean number of alleles) with rarefaction; *N*_E_, mean number of effective alleles; *H*_o_, mean observed heterozygosity; *H*_e_, mean expected heterozygosity; *F*_IS_, mean fixation index; *N*_hp_, number of chloroplast haplotypes; *H*_cp_, mean gene diversity index for haplotype. Significance level was marked as followings: ^ns^*P* > 0.05, *P < 0.05, **P < 0.01. SD stands for standard deviation.

Locality	Acronym	Nuclear microsatellite							Chloroplast	
									*psb*A–*trn*H	
		*N*	*P*	*N*_A_ [±SD]	*N*_E_ [±SD]	*H*_o_ [±SD]	*H*_e_ [±SD]	*F*_IS_	*N*_hp_	*H*_cp_ [±SD]
Mt. Cheongryang	CR	187	80	3.13 [1.80]	1.79 [0.75]	0.29 [0.15]	0.35 [0.25]	0.188**	2	1 [0.006]
Mt. Choijeong	CJ	63	80	2.21 [0.95]	1.21 [0.25]	0.15 [0.11]	0.14 [0.16]	0.140*	1	1 [0.002]
Mt. Jiri Baengmu	BM	30	100	3.39 [1.96]	2.21 [1.35]	0.35 [0.17]	0.43 [0.23]	0.184**	2	1 [0.008]
Mt. Jiri Chilseon	CS	30	100	3.00 [0.63]	2.18 [0.20]	0.40 [0.17]	0.54 [0.05]	0.272**	2	1 [0.009]
Mt. Jiri Ungseok	US	90	100	4.83 [1.16]	1.39 [0.31]	0.22 [0.20]	0.24 [0.19]	0.117**	1	1 [0.001]
Mt. Hogu	HG	48	80	2.98 [0.63]	1.38 [0.43]	0.29 [0.30]	0.21 [0.24]	−0.107^ns^	2	1 [0.004]
Mt. Mangun	MU	31	60	3.00 [0.75]	1.52 [0.57]	0.39 [0.22]	0.25 [0.28]	0.059^ns^	1	1 [0.008]

### Microsatellite amplification

Five microsatellite markers (Table 1) developed by Yun *et al.* (2015) were used to genotype the 479 individuals from seven populations. We tested for possible problems with null alleles using MICRO-CHECKER 2.2 (Van Oosterhout *et al.* 2004). PCRs were performed in a 15 μL volume containing 30–50 ng of template DNA, 0.5 μL dNTPs (20 mM), 1 μL 10× PCR buffer containing 25 mM MgCl_2_ (TAKARA, Japan), 0.25 μL forward and 1 μL reverse primers (8 pmol each), and 1 μL fluorescently labelled M13 primer (8 pmol; 6-FAM, VIC, PET and NED). PCR cycling conditions were as follows: 5 min pre-denaturation at 94 °C followed by 30 cycles of 30 s at 94 °C, 45 s at 56 °C and 45 s at 72 °C, followed by eight cycles of 30 s at 94 °C, 45 s at 53 °C and 45 s at 72 °C, and then a final 20 min extension step at 72 °C. The fluorescently labelled PCR products were pooled with a loading buffer of Hi-Di™ formamide (Applied Biosystems, USA) and a size standard GS500LIZ (Applied Biosystems, USA). Amplified fragments were then separated out on an ABI 3730XL automated sequencer (Applied Biosystems, USA). The results of the microsatellite profiles were examined with GeneMarker program (version 2.40, Softgenetics LLC) using automated scoring and manual double-checking.

### Chloroplast DNA polymorphism

Based on preliminary laboratory screening, we chose the chloroplast intergenic spacer, *psbA-tranH * (*psbA-tranH* IGS). Amplifications of the selected region were performed in a final reaction volume of 20 µL, containing 10 ng genomic DNA, 10 pmol each of primer and PCR Premix Accupower PCR Premix (Bioneer, USA). We conducted PCR amplification using primer sets as in Sang *et al.* (1997) with the following conditions: initial denaturation at 95 °C for 3 min; 35 cycles of 1 min at 95 °C, 1 min at 54 °C and 1 min at 72 °C; and a final extension at 72 °C for 7 min. The size of the PCR products was verified on 1 % agarose gels. The amplified products were analysed using an ABI 3730XL DNA Analyzer and the target sequences were checked for correct amplification using Sequencer version 5.0 (Gene Codes Corp, Ann Arbor, MI, USA).

### Data analysis

We calculated the following genetic diversity estimates in GeneAlex 6.5 (Peakall and Smouse 2012): percent of polymorphic loci, allelic richness (*N*_A_), expected heterozygosity (*H*_e_) and observed heterozygosity (*H*_o_). Allelic richness (*N*_A_) was calculated using rarefaction curves to standardize across seven populations to 60 genes (Table 1; Kalinowski 2004) in HP-Rare (Kalinowski 2005). Pairwise *F*_ST_ between all seven population pairs were calculated based on five microsatellite markers in Arlequin version 3.5 with 1000 permutations for the significance test (Excoffier and Lischer 2010). We used *F*_ST_ measures instead of *R*_ST_, divergence metric specifically designed for microsatellite markers, because *R*_ST_ is the only unbiased and reliable metric under a strict stepwise mutation model, which is unrealistic (Meirmans and Hedrick 2011). Analysis of molecular variance (AMOVA) was conducted to test geographic structure in Arlequin version 3.5 (Excoffier and Lischer 2010). The re-sampled data of 1000 permutations were compared to test statistical significance. We performed the Mantel test to test for isolation by distance (IBD) using pairwise genetic divergence (*F*_ST_) and Euclidean distance for 21 population pairs in GENALEX 6.5 (Rousset 1997; Peakall and Smouse 2012). Statistical significance was tested using 1000 random permutations with replacement.

We assessed departure from Hardy–Weinberg equilibrium (HWE) and pairwise linkage disequilibrium (LD) for each locus in Arlequin version 3.5 (Excoffier and Lischer 2010). Because most of our molecular markers were neither in HWE nor independent from each other, discriminant analysis of principal components (DAPC) was performed in ‘adgenet’ R package (Jombart and Ahmed 2011) instead of the most common clustering approach, STRUCTURE. Discriminant analysis of principal components analysis is a multivariate algorithm, similar to principal component analysis (PCA) that identifies genetic clusters (Jombart *et al.* 2010). Unlike STRUCTURE, DAPC does not hold for any assumptions such as HWE or LD, which makes it a better approach for assignment analysis with problematic markers than STRUCTURE (Jombart *et al.* 2010). To infer the best *K*, the number of subgroups genetically related, we ran the analyses with different numbers of clusters (*k* = 1 through 7), and chose the best *K* model based on Bayesian information criterion (BIC). To avoid overfitting, we calculated the optimal number of PCs to be retained for DAPC analysis using function ‘optim.a.score’ in R package ‘adgenet’ (Jombart and Ahmed 2011; R Core Team 2016).

Population at mutation-drift equilibrium approximately shows an equal probability of heterozygosity excess or heterozygosity deficit. To examine whether a population has experienced recent bottlenecks, we tested for excess of heterozygosity as described in Cornuet and Luikart (1996) using the software BOTTLENECK (Piry *et al.* 1999). The significance between observed and modelled heterozygosity within each population was tested under infinite allele model with Wilcoxon signed-rank test accounting for issue with small number of markers.

The sequence fragments obtained in this study were aligned with GENETIX-WIN ver. 4.0.1 (Software Development, Tokyo, Japan) to identify sequence variants. The integrated software package DnaSP version 5 (Librado and Rozas 2009) was used to determine the haplotypes. We constructed a haplotype network to determine relationships among the four unique haplotypes found from chloroplast marker, *psbA-trnH* IGS, using 95 % statistical parsimony (Templeton *et al.* 1992) with TCS algorithm (Clement *et al.* 2000) implemented in the software popart 1.7 (http://popart.otago.ac.nz). Because of the non-recombining nature of the chloroplast genome, chlorotypes were then treated as alleles at a single locus. Gene diversity index (*H*_cp_), equivalent to the expected heterozygosity for diploid data, was calculated using ARLEQUIN version 3.5 (Excoffier and Lischer 2010) using the Nei’s formula (Nei 1987).

## Results

### Genetic diversity and population bottleneck

Allelic richness (*N*_A_) and number of effective alleles (*N*_E_) showed different patterns across the populations investigated (Table 1). *N*_E_ was the lowest at Choijeong (1.21) and the highest at Chilseon (2.18), whereas *N*_A_ was the lowest at Mangun (1.80) and the highest at Baengmu (3.39) (Table 1). Likewise, expected heterozygosity (*H*_e_) within populations ranged from 0.143 (Choijeong population) to 0.538 (Chilseon population; Table 1). The rest of the genetic diversity estimators showed a similar pattern (Table 1). The inbreeding coefficients were significantly different from zero for the most of populations except for the two populations, Hogu and Mangun (Table 1). The inbreeding coefficient was higher at Chilseon population where the size of population is much smaller (consensus population size, ca. <30) than the rest populations (Table 1). Results of BOTTLENECK indicated that there is a significant excess of heterozygotes in Chilseon population, whereas the remaining six populations did not exhibit the same pattern (Wilcoxon signed-rank test, *P* < 0.05 under infinite allele model).

### Population structure

Thirteen of the 35 tests (7 populations * 5 five loci) exhibited significant deviations from HWE (*P* < 0.05). There was no evidence of null alleles in any of five loci after correcting for missing values. Significant LD was observed among 5 of the 10 locus pairs (*P* < 0.05 using Fisher’s exact test). Overall pairwise population divergences (*F*_ST_) were high across all seven populations of *A. austrokoreense* in South Korea, particularly in four populations (BM, HG, MU, US; see Table 1 for population abbreviations) near Mt. Jiri have diverged greatly from central part of South Korea (CJ and CR; Table 2). Analysis of molecular variance showed that 51 % of genetic variation is within populations and that 39 % of the variation is partitioned to among-population genetic variations (Table 3). *F*_ST_ averaged over all population pairs was 0.39 based on the five microsatellite loci (Table 2).

**Table 2.** Pairwise *F*_ST_ among seven *Aconitum austrokoreense* populations in South Korea. All *F*_ST_ values presented were significant at *P* < 0.05. See Table 1 for population abbreviation. Grand mean = mean of pairwise *F*_ST_ across all population pairs.

	CR	CJ	BM	CS	US	HG	MU
CR	0						
CJ	0.35	0					
BM	0.34	0.41	0				
CS	0.30	0.55	0.22	0			
US	0.42	0.44	0.16	0.43	0		
HG	0.46	0.54	0.11	0.43	0.32	0	
MU	0.46	0.54	0.05	0.38	0.24	0.03	0
Grand mean 0.39							

**Table 3.** Results of AMOVA. All variance components were statistically significant (*P* < 0.005); df stands for degrees of freedom.

Source	df	Sum of squares	Percentage of variation	Fixation index
Among populations (*F*_ST_)	6	360.117	39	0.385
Among individuals (*F*_IS_)	472	422.821	11	0.171
Within individuals (*F*_IT_)	479	303.500	51	0.490

**Figure 1.**
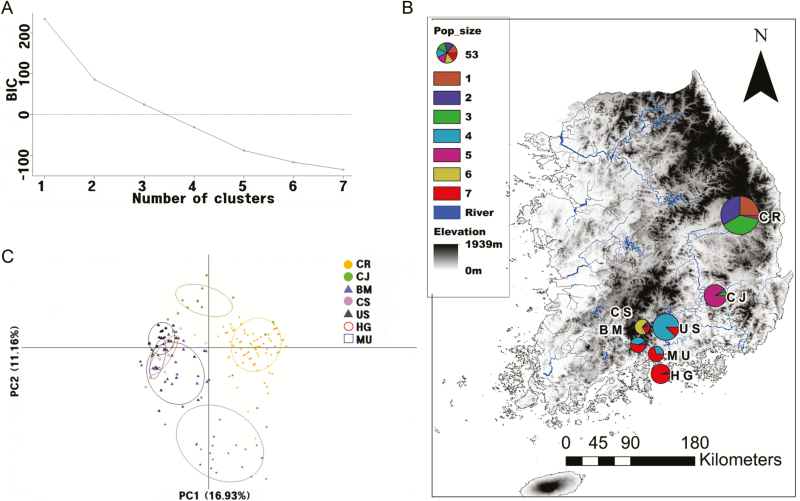
Discriminant analysis of principal components (DAPC)-based clustering analysis for seven populations of *Aconitum austrokoreense* in South Korea. (A) Based on BIC, seven genetically distinct groups were identified (*K* = 7); (B) PCA scatterplot of the seven *A. austrokoreense* populations collected throughout South Korea. The axes are the first two components explaining maximum genetic structure; (C) Pie charts show DAPC analysis-based group assignment for individuals in each population across South Korea. The sizes of the pie charts indicate the relative sample size.

Non-parametric clustering analysis, DAPC analyses, showed the highest support for *K* = 7 based on the BIC value (Fig. 1A). Discriminant analysis of principal components of five microsatellite markers clearly indicated that the clustering patterns of genotypes are consistent with their geographic origins (Fig. 1B). Four populations (BM, HG, MU, US; see Table 1 for population acronyms) around Mt. Jiri shared genetic similarity, whereas CS population is genetically more distant from the four populations despite the short geographic distance (Fig. 1C). CJ and CR populations showed different genetic compositions from the southern populations near Mt. Jiri (Fig. 1C). Consistent with the spatial pattern inferred from the DAPC results, there was significant correlation between pairwise geographic (Euclidean) distance and *F*_ST_ (Mantel test; *r* = 0.48, *P* < 0.05), suggesting a pattern of IBD. We also performed the Mantel test with Rousset’s (1997) linearized *F*_ST_ [i.e. *F*_ST_/(1 − *F*_ST_)]; however, the transformation was not suitable as the *F*_ST_ values were normally distributed and appeared to have stronger correlations with the Euclidean distance than did the linearized *F*_ST_.

### Chloroplast haplotypes and diversity pattern

**Figure 2.**
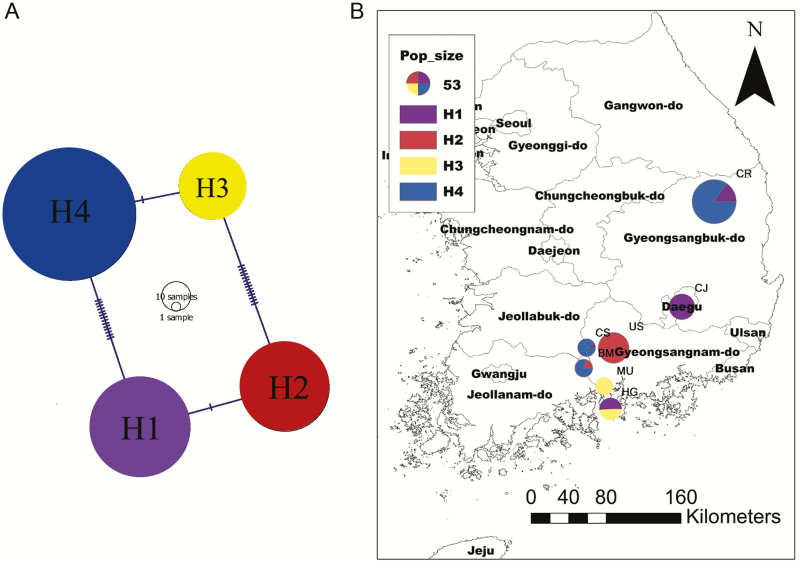
Chloroplast *psb*A–*trn*H IGS haplotype network of 479 *Aconitum austrokoreense* in South Korea. (A) A diagram shows haplotype network among seven populations. Lines separating the haplotypes represent a single point mutation or insertion/deletion event. (B) Pie charts represent contribution of four haplotypes in each population. The circle size is proportional to frequency of each haplotype.

The chloroplast *psb*A–*trn*H region was successfully amplified and aligned for length of 271 bp including 15 nucleotide variations (DNA sequences: GenBank accessions MH078578–MH079047). We identified four haplotypes (H1–H4; 15 mutational steps) from 479 individual plants in seven populations throughout South Korea (Table 1; Fig. 2). The haplotype network (Fig. 2) depicts relative frequency of each haplotype and mutational changes between haplotypes. The predominant haplotype was Haplotype 4 (frequency, 0.44), which mainly occurred in three populations (CR, BM and CS; see Table 1 for population abbreviation; Fig. 2). Interestingly, Haplotype 2 was present in three populations along Mt. Jiri with significantly high frequency (frequency of 1) in the Ungseok population (Fig. 2). Haplotype 1 (frequency, 0.25), the second most frequent haplotype, was present in four populations (CR, CJ, CS and HG). Haplotype 3 (0.11) was rare and only occurring seashore in southern part of South Korea peninsula (MU and HG). The gene diversity for chloroplast haplotypes (*H*_cp_) was 1 for all seven populations (Table 1).

## Discussion

As populations become small, population growth and persistence is highly influenced by stochastic events such as genetic drift and inbreeding resulting in reduced genetic diversity and fitness (Frankel and Soulé 1981; Ellstrand and Elam 1993; Ouborg *et al.* 2010). Endangered plant species often exhibit a narrow and isolated geographic distribution. Therefore, genetic diversity is expected to be lower than plant species that have wide geographic range with large population size (Hamrick *et al.* 1991; Ouborg *et al.* 2010). Overall, like many rare and endangered plant species, *A. austrokoreense* showed limited genetic variation particularly in allelic richness (Table 1). The allelic richness is significantly lower than average allelic diversity (~10 alleles for a locus) observed in many other genetic studies of microsatellite loci (Nybom 2004). Interestingly, compared to allelic diversity, heterozygosity has been relatively well maintained (*H*_e_, ranged from 0.14 to 0.53). Alleles are more vulnerable to be lost when there is abrupt reduction in population size (Nei *et al.* 1975). The stronger loss of allelic richness we observed in *A. austrokoreense* may be the result of drastic decline in population size (i.e. recent bottlenecks). In fact, our analysis of BOTTLENECK showed that there was a significant excess of heterozygosity for CS population (Wilcoxon signed-rank test, *P* < 0.05), which is consistent with recent bottleneck. Although it was only marginally significant in statistical analysis, the rest populations also showed signs of recent bottlenecks (Wilcoxon signed-rank test, 0.05 < *P* < 0.1).

**Figure 3.**
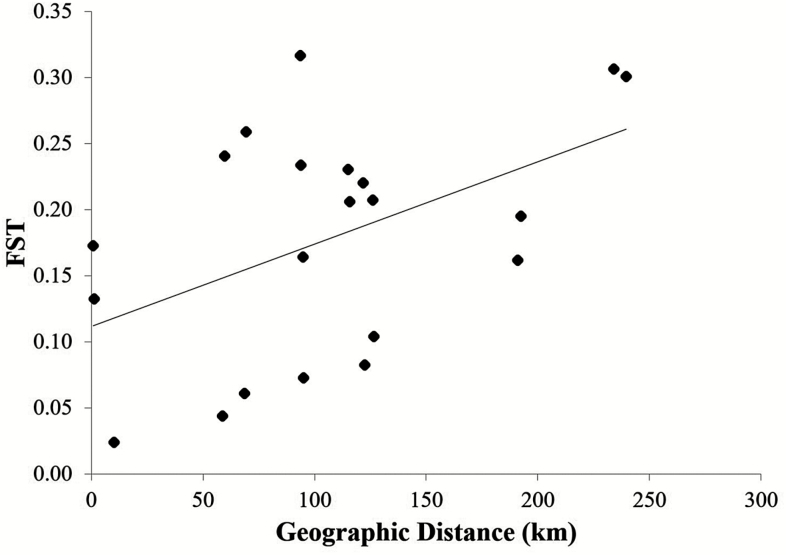
The correlation between geographic distance and *F*_ST_ for 21 *Aconitum austrokoreense* population pairs from South Korea. A significant IBD pattern was identified through a Mantel test (*r* = 0.48, *P* < 0.05).

Additionally, high positive *F*_IS_ values found in five of seven populations suggest possible inbreeding in those populations in part due to population isolation and lack of habitat connectivity (Table 1). Inbreeding may also explain the significant LD we found in three neighbouring populations along Mt. Jiri and the deficiency of heterozygotes, likely the cause of significant deviation from HWE. Discontinuity among habitats was further supported by pairwise *F*_ST_ (Table 2). *F*_ST_ is an indirect estimator of population connectivity among subpopulations (Wright 1943; Lowe and Allendorf 2010). More than half of pairwise *F*_ST_ values we assessed were greater than 0.35 which were higher than average microsatellite-based *F*_ST_ for perennial herbs (short lived = 0.31; long lived = 0.19; Nybom 2004). As shown in increased *F*_ST_ values, gene flow among the populations of *A. austrokoreense* might be very limited leading to accumulation of harmful effects of inbreeding in isolated populations with high inbreeding rate (Lowe and Allendorf 2010). A significant pattern of IBD (Fig. 3) also supports limited gene flow. In general, population pairs that are the most geographically distant were the ones that are genetically the most diverged.

Discriminant analysis of principal components results showed that three neighbouring populations (BM, US and CS) along Mt. Jiri with dense forests were more tightly clustered from the rest central populations (CR and CJ; see Table 1 for acronyms; Fig. 1). The large-scale spatial pattern of population structure identified by DAPC results clearly showed strong influence of mountain ranges on population divergence in *A. austrokoreense*. Dense forests and high mountains may largely contribute to environmental differences for each of populations and offer physical barriers for bee pollination. Consistent with the pattern found in microsatellite variation, the geographic pattern of chloroplast haplotype variation also separated out the two central populations from the southern populations (Cheongryang and Choijeong; Fig. 2). However, chloroplast haplotype variation exhibited more complex clustering patterns among populations. For example, southern populations near the sea shore clearly differ from the rest southern populations (Fig. 2). Different inheritance modes of genes (cytoplasmic vs. nuclear) influence the pattern of genetic structure (Petit *et al.* 2005). Because chloroplast genes are mostly inherited maternally in angiosperms, gene flow in chloroplast genes is restricted to seed movement, whereas bi-parentally transmitted nuclear genes can migrate by either seeds or pollen dispersal (McCauley 1994). The complexity of population structure found in chloroplast haplotype variation may be the result of more limited dispersal ability of *A. austrokoreense* seeds rather than pollen dispersal by bees.

## Conclusions

One of the primary goals for conservation genetics is to understand patterns of genetic diversity and ultimately identify conservation units that are genetically similar. Our study revealed that populations of *A. austrokoreense* have the limited genetic variability and high genetic structure, which might be detrimental to species persistence, as the species may be more vulnerable to novel selection pressure (Ellstrand and Elam 1993; Dlugosch and Parker 2008; Sheth and Angert 2014). Human influences such as habitat destruction and poaching may have been the major cause of limited gene flow and genetic variation for the species. However, human influences may be more complex than expected as they might have contributed to long-distance migration of the species. For example, the two distant populations (CR and CJ; see Table 1 for acronyms) represented by Haplotype 1 and 4 (Fig. 2) may have been introduced to the area from Mt. Jiri, the main habitat by human collection due to its medicinal values although current data set cannot empirically test the hypothesis.

Nevertheless, in the light of population connectivity, ongoing habitat destruction could lead to even more increased isolation between populations for the species. For management practices, we propose that maintaining connectivity of the small habitats within the scattered localities and constant monitoring for *A*. *austrokoreense* would be of great help. For example, neighbouring populations around Mt. Jiri that share high genetic similarity may well be a conservation unit, whereas the other two populations in the central part of South Korea (CR and CJ; Figs 1 and 2) may be treated separately for conservation plans. Finally, vast effort should also be made to investigate mating systems and dispersal modes of the species given the importance of those factors on genetic diversity (Loveless and Hamrick 1984; Swift *et al.* 2016).

## Sources of Funding

This work was supported by the grant, NIBR201503101, from the National Institute of Biological Resources, Republic of Korea.

## Contributions by the Authors

C.E.L. and B.-Y.L. designed the project and arranged supporting grant. Sample collections, laboratory work and microsatellite genotyping were conducted by J.-E.C. and J.-N.Y. S.-R.L. conducted genetic and related statistical analyses and wrote the manuscript. C.E.L. and S.-R.L. conceived ideas and revised the manuscript. All authors edited the manuscript and agreed to submit current version of manuscript.

## Conflict of Interest

None declared.
